# The Concept of Clinical Economics and its Relation with Effectiveness

**DOI:** 10.5935/abc.20170084

**Published:** 2017-06

**Authors:** Franz Porzsolt, Luis C. L. Correia

**Affiliations:** 1 Pesquisa em Cuidados de Saúde - Departamento de Cirurgia Geral e Visceral - Hospital Universitário de Ulm, Alemanha; 2 Instituto de Economia Clínica, Ulm - Alemanha; 3 Escola de Medicina e Saúde Pública da Bahia, Salvador, BA - Brazil; 4 Hospital São Rafael, Salvador, BA - Brazil

**Keywords:** Clinical Trials as Topic / analysis, Cost-Benefit Analysis, Treatment Outcome

Clinical Economics should be an essential component of medical education and practice. In defining Clinical Economics, we should make clear that economical thinking is not primarily a monetary issue.

A classical economic analysis considers four aspects: first, the costs, i.e. what somebody has to give away; second, the consequences, i.e. what somebody gets back; third, the comparison of the relation of both costs and consequences of alternative ways of actions; fourth, the perspective of the person who makes the economic analysis.

To provide an example we start with the perspectives. From a patient’s perspective, the alternative ways of action may be either immediate surgery or watchful waiting if there is a realistic chance of spontaneous regression. The costs for the patients will be an increased risk of complications in case of watchful waiting. The consequence (advantage for the patients) in this situation is the chance to avoid surgery. The perspective of the hospital manager will be different. He will also consider costs and consequences, but of different types, such as monetary costs and monetary consequences. Doctors and managers of a hospital have to do different jobs and to make different decisions. In some places the same person is responsible for both decisions. This is like somebody who is playing chess against himself.

Economic decisions are based on values, and values are different in different people. Clinical Economics is focusing on the from the perspectives of patients and doctors, but not from the perspectives of managers. It is obvious that no hospital will survive and no healthcare system will be affordable unless the perspectives of economists, managers and politicians will be considered. The difficult consensus process among people with different perspectives and values is shown in Figure 1.

**Figure 1:**
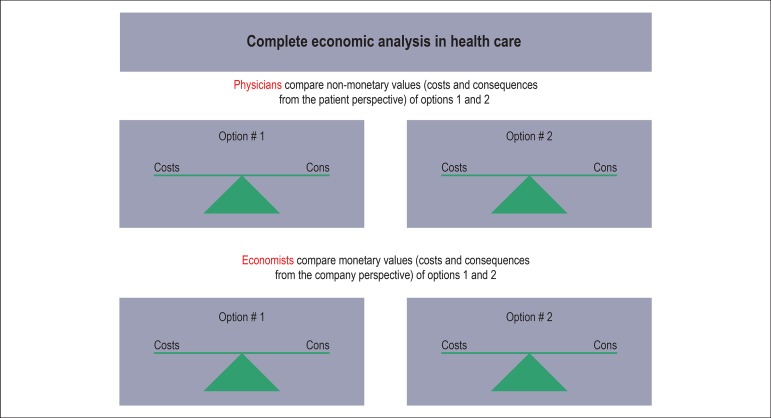
The complete economic analysis includes costs and consequences of different options. In medicine there are two important parties who’s perspectives have to be considered and combined: frst, the individual perspectives of the patients and their doctors and second, the natural perspectives of the patient without or with surgical operation.

Before thinking monetarily, physicians and patients need to figure out how much they have to give away (the costs) and what they get back (the consequences or benefit). Clinical investment is the “cost” a patient pays for accepting a treatment, such as pain, side effects, time spent, possible adverse events, or psychological distress. The “profit” is the value the patient gets back from his or her investment. Usually physicians do not consider this trade off, disregarding how much is the investment and overestimating the returned value (effect size). In addition, price and profit vary according to patient preferences. The value a pianist takes from a surgical repair of his or her hand is much higher than the value a lawyer would take back from the same surgery, because the proper function of the hand is more important to the first.

Clinical Economics is about efficiency, which can be defined as a cost-effective trade-off. A key moment in the history of Clinical Economics was the question of my teacher at the Ontario Cancer Institute in Toronto/Ontario about the German word for ‘efficiency’. He was amused when I mentioned the word “Effizienz” in my response to him. He concluded the word ‘efficiency’ does obviously not exist in the German language. This terrible conclusion was a real strong motivation to demonstrate what efficiency means from a German perspective. Our group started to clarify the difference of efficacy and effectiveness and its relation to efficiency that was by far not as clear 20 years ago as it is today.

## Efficacy versus Effectiveness

We underlined that efficacy and effectiveness describe two different types of information: efficacy is the demonstration that a new principle can theoretically work, which comes from studies under ideal controlled conditions; effectiveness is how the concept proven by efficacy studies works under real world conditions (RWC).^1,2^ For demonstration of efficacy, one should select the optimal scenario for the proof of principle. It requires an experimental study design, with random allocation of treatments, to eliminate confounding bias and proper assessment of causality, it means the trial is explanatory.

The demonstration of effectiveness is pragmatic and takes place in the scenario in which the new principle will be used (RWC). The design is observational and treatment allocation is under the discretion and preferences of physician and patient. It allows assessment of the two main determinants of effectiveness: practical issues regarding adequate applicability of treatment and the impact of individualized choices. The interaction of these two forces determines whether the effectiveness of a treatment will be smaller than its proven efficacy (loss of beneficial effect in the real world) or whether the treatment will be even more effective than efficacious. The first situation should be a concern when logistic issues impair the treatment to be ideally applied (a not well trained doctor, a patient not educated enough to properly take an anticoagulant, a system not able to provide adequate door-to-balloon time in primary angioplasty for acute myocardial infarction), what tends to happen when the treatment is somewhat complex. The second situation takes place when physicians and patients provide a better solution than a simple randomization can do allocation.

## The actual effectiveness study

A pragmatic controlled trial (PCT), but not a randomized control trial (RCT), should be used for demonstration of effectiveness as a RCT can never reflect RWC.^3^ To understand the contribution of PCTs to the existing RCTs we list the differences of these two trials:

1. Instead of randomization, the patients are stratified in a PCT to different risk and treatment groups.
2. The factors that characterize the risk groups are selected before start of the trial for each of the study endpoints.
3. A PCT can investigate multiple primary endpoints, e.g. mortality, specific aspects of quality of life, and cost of care, while a RCT can investigate only a single primary endpoint, but several secondary endpoints. This secondary endpoint cannot confirm or reject a hypothesis, but may generate new hypotheses.
4. The individual risks of the included patients are known in a PCT, but not in a RCT. The efficacy observed in a RCT reflects the average efficacy only related to the mix of risks in the investigated group. In a PCT, the effectiveness is described separately for each endpoint, for each risk group and for each treatment group.
5. A PCT uses inclusion, but no exclusion criteria, because a patient who meets the inclusion criteria cannot be excluded from care which may sometimes be ‘wait and see’ under RWC.
6. A PCT is a descriptive study in contrast to a RCT which is an explanatory study. Power calculation is not possible in a descriptive study as neither sample size, nor effect size, nor alpha-error and beta-error are known prospectively.
7. The approval by an institutional review board is necessary in a PCT for systematic collection and for publication of patient data.
8. An intent-to-treat analysis is not necessary in a PCT, as the patients cannot change the allocation to the risk group even if the treatment strategy is changed in the ongoing study.
9. The calculation of the statistical significance is not necessary for results that are clinically irrelevant. Statistical confirmation of a clinically irrelevant result is a waste of statistical power.

The bottom-line message is that RWC are essential for making reliable clinical decisions. The results obtained by RCTs under ideal world conditions are essential to justify the use of a new intervention under RWC. In addition, we need the effectiveness and the efficiency under RWC to justify a new intervention in recommendations and clinical guidelines.

We are not expecting that the described tools developed with several colleagues in the last decade^4,5^ offer optimal solutions, but we hope that the offered tools and strategies will trigger a discussion on the further development of this growing discipline.

## Blindness to effectiveness and overuse

The problem of *overuse* was recently addressed in *The Lancet* as one of the important challenges of the next decade.^6,7^ *Overuse* takes place when useless tests or treatments are utilized, leading to *overdiagnosis* or *overtreatment. Overuse* is typically related to lack of efficacy. We believe the concept of *overuse* should be expanded beyond efficacy. An efficacious treatment not properly tested for effectiveness is also at risk to be an *overtreatment*. However, physicians are normally blind to effectiveness, missing the need of test for it. Especially in situations in which applicability of the treatment is complex, effectiveness studies should be mandatory to avoid *overuse*. Among other important steps in the development of evidence-based Medicine, the addition of PCTs to the existing RCTs may be an important development.
